# Eating disorders risk assessment and body esteem among elite polish contact karate athletes

**DOI:** 10.3389/fnut.2025.1704743

**Published:** 2025-11-25

**Authors:** Daria Dobkowska-Szefer, Wiktoria Staśkiewicz-Bartecka, Agata Kiciak, Marek Kardas

**Affiliations:** Department of Food Technology and Quality Evaluation, Department of Dietetics, Faculty of Public Health in Bytom, Medical University of Silesia, Katowice, Poland

**Keywords:** eating disorders, orthorexia nervosa, body image, body esteem, EAT-26, contact karate, elite athletes

## Abstract

**Background:**

The prevalence of disordered eating behaviors and body image disturbances among athletes in weight-category sports remains insufficiently explored, especially in combat disciplines such as contact karate. The present study aimed to assess the risk of eating disorders and body image perception among elite Polish contact karate athletes, considering the influence of competitive pressure, weight-category requirements, and body image ideals on nutritional behaviors and mental health.

**Methods:**

This cross-sectional descriptive study involved 55 elite contact karate athletes, all with at least one medal at the Polish Championships or higher level competitions. Data were collected using a computer-assisted web interview (CAWI) and validated tools: the Eating Attitudes Test (EAT-26), the Düsseldorf Orthorexia Scale (DOS-PL), and the Body Esteem Scale (BES).

**Results:**

Applying all three EAT-26 referral criteria, 58.12% of athletes threshold indicating potential eating-disorder risk; 12.73% screened positive on the EAT-26 symptom score (Part A), which evaluates attitudes toward eating and dieting behaviors, reflecting one’s relationship with food. On DOS-PL, 10.9% met the orthorexia cut-off and a further 20% were at elevated risk. Weight-making behaviors were common 47.47% reported cutting weight, typically ~4.6% body mass per event. Sex differences emerged: women scored higher on EAT-26 Part A (*p* = 0.021) and reported lower body-esteem in sexual attractiveness and weight control, whereas men most often reported medium physical attractiveness with frequent low ratings for upper-body strength/physical condition. In women, higher body-esteem correlated with lower EAT-26 totals, and EAT-26 correlated positively with DOS-PL (*r* = 0.367, *p* = 0.036); these associations were not observed in men.

**Conclusion:**

The finding that over half of elite contact karate athletes exhibit risk indicators for eating disorders highlights the urgent need for systematic screening and intervention in weight-category sports. Prevention programs should integrate nutritional education, mental health support, and early identification strategies to protect both performance and long term health.

## 1 Introduction

Karate is a martial art based on the skills of strikes, punches, and kicks. It is traditionally divided into three components: kihon (basic techniques), kata (predetermined sequences of offensive and defensive movements), and kumite (sparring between two athletes). Depending on the style and governing organization, kumite may involve varying rules, ranging from light contact to full contact combat. During competitions, athletes are divided into weight categories (1). Recreational sport is associated with numerous benefits, including increased self-confidence, improved body image, and the promotion of healthy eating habits (2–5). However, competitive sport introduces the pressure of performance, which is accompanied by both psychological and physical strain (6–8). Requirements related to body weight and appearance heighten the risk of eating disorders, particularly in sports where these factors play a pivotal role in performance outcomes (9–12). According to the ICD-11 classification, eating disorders include anorexia nervosa, bulimia nervosa, avoidant/restrictive food intake disorder (ARFID), pica, rumination-regurgitation disorder, other specified eating disorders, and unspecified eating disorders (13). Anorexia nervosa is characterized by severe dietary restriction, often combined with purging behaviors. ICD-11 also recognizes atypical forms of anorexia, in which certain symptoms (e.g., normal body weight) are less evident but nonetheless lead to significant health consequences. Bulimia nervosa involves dietary restriction alternating with recurrent binge eating episodes, followed by compensatory behaviors such as self-induced vomiting, use of laxatives or diuretics or excessive exercise. Binge-eating disorder consists of uncontrolled eating episodes without compensatory behaviors, often accompanied by shame and a sense of loss of control. ARFID involves food avoidance not related to distorted body image (11, 13). Pica refers to the ingestion of non-nutritive substances (e.g., soap). The category of other specified eating disorders encompasses conditions that do not fully meet the criteria of the above but cause substantial health or social impairment. Unspecified eating disorders include emerging conditions such as orthorexia (13). Orthorexia refers to a pathological preoccupation with consuming exclusively “healthy” foods (14, 15). Although not formally recognized in the DSM-5 or ICD-11, increasing evidence suggests its detrimental impact on athletes’ physical and psychological wellbeing (16, 17). The absence of formal diagnostic criteria in international classifications makes orthorexia challenging to identify. In 2022, an international consensus of experts proposed unified, though still unofficial, diagnostic criteria for orthorexia, underscoring its potential health risks and the necessity of further research (18).

The etiology of eating disorders is multifactorial, involving genetic, environmental, and behavioral components (6–8). Importantly, body image perception plays a critical role in their development. The pursuit of an ideal body, often reinforced by the sports environment and the media, may lead to body dissatisfaction, low self-esteem, and maladaptive eating behaviors (7, 9). Karate, as a discipline that combines sport, martial art and philosophy, imposes high physical and psychological demands on its practitioners. Competitive participation is organized by weight categories, which frequently requires athletes to maintain or rapidly reduce body weight prior to events (1). Repeated engagement in such practices, particularly in the absence of professional guidance, may foster disordered eating patterns and distorted body image. Combined with performance pressure and perfectionism, these factors heighten the risk of eating disorders, including anorexia, bulimia, and orthorexia (9, 11).

The assessment of body image, dietary behaviors, and potential orthorexia symptoms in this population may provide valuable insights into psychological risks in sport. Moreover, it may guide the development of preventive and educational strategies targeted at athletes, coaches, and caregivers. Early identification of these issues is crucial not only for preserving athletes’ physical and mental health but also for ensuring their long-term athletic development (10, 12, 19). The aim of the present study is to examine the relationship between the risk of eating disorders, including orthorexia, and body image perception among athletes practicing contact karate. Additionally, the study seeks to determine whether factors such as body mass index (BMI) and weight reduction practices influence this relationship. It was hypothesized that athletes with lower body image evaluation would present a higher risk of eating disorders and orthorexia, and that individuals at risk of these disorders would more frequently demonstrate lower BMI values or engage in pre-competition weight reduction behaviors.

## 2 Materials and methods

### 2.1 Procedure

The study followed a cross-sectional descriptive design aimed at identifying associations between eating attitudes, orthorexic tendencies, and body esteem among elite contact karate athletes. The study was conducted between November 2024 and December 2025. Participants were recruited from contact karate clubs and were required to have achieved at least the level of the Polish Championships. Data were collected using the Computer Assisted Web Interview (CAWI) method. The study was carried out via an online questionnaire which is an accepted tool in psychological research. The Google Forms platform was chosen due to its accessibility and ease of use.

Recruitment was conducted through direct contact with karate clubs, which were informed of the purpose and details of the study. Clubs were asked to indicate athletes who met the inclusion criteria, and a link to the questionnaire was subsequently sent directly to eligible participants.

All athletes were informed of the study’s aims and anonymity, and were asked to provide consent for data collection. Information on voluntary and informed participation was provided at the beginning of the questionnaire. The study complied with the Declaration of Helsinki of the World Medical Association and was approved by the Bioethics Committee of the Medical University of Silesia in Katowice (BNW/NWN/0043-3/641/35/23, approval date: September 22, 2023), in accordance with the Act of December 5, 1996, on the Profession of Physician and Dentist (Journal of Laws 2016, item 727).

### 2.2 Participants

The study involved athletes training in contact karate in Poland, representing three main styles: Kyokushin, Shinkyokushin, and Oyama. Participants were members of clubs located across the country. A total of 56 athletes initially met the inclusion criteria, defined as: (1) consent from both the club and the athlete to participate in the study, (2) active participation in training in contact karate clubs (defined as ≥3 training sessions per week for at least the past 6 months), (3) achievement of at least one medal-winning position in competitions at the level of the Polish Championships or higher, organized by national or international sports federations, and (4) absence of chronic diseases, including diagnosed mental health disorders, as confirmed in the demographic section of the questionnaire. The exclusion criteria were defined as: (1) age below 18 years, (2) incorrectly or incompletely completed questionnaires. One athlete was excluded from the analysis; thus, the final sample comprised 55 contact karate athletes aged 19–49 years. Participants were divided into two groups according to sex: the first group consisted of women (*n* = 33), and the second group consisted of men (*n* = 22).

### 2.3 Research instruments

The questionnaire included a demographic section with items on age, height, body weight, chronic diseases, medication use, education, food exclusions, competitive weight category, weight-cutting behaviors, weekly training hours and sources of nutritional knowledge. In addition, the following standardized instruments were applied: Eating Attitudes Test (EAT-26), Düsseldorf Orthorexia Scale (DOS-PL), Body Esteem Scale (BES).

### 2.4 Body mass index (BMI)

To assess the nutritional status of the participants, the Body Mass Index (BMI) was calculated using the standard formula:

$\mathrm{BMI}=\frac{\text{body weight}\;[\mathrm{kg}]}{\text{height}\;{\left[m\right]}^{2}}.$

The results were interpreted according to the guidelines developed by the World Health Organization (WHO), ensuring consistency with international standards. A detailed summary of the data is presented in Table 1.

**Table 1:** BMI classification according to WHO (33).

BMI (kg/m^2^)	BMI interpretation
<18.5	Underweight
18.50–24.99	Normal body weight
25.00–29.99	Overweight
30.00–34.99	First-degree obesity
35.00–39.99	Second-degree obesity
≥40.00	Third-degree obesity

### 2.5 Eating attitudes test (EAT-26)

The Eating Attitudes Test-26 (EAT-26) is a standardized screening tool designed to assess attitudes toward eating and the risk of developing eating disorders. It was developed in 1982 by Garner (20). The Polish standardization of the instrument was conducted by Włodarczyk-Bisaga and Dolan (21). The test was created to screen both individuals with a clinical diagnosis and those at risk of developing anorexia nervosa, bulimia nervosa, binge eating disorder, and atypical forms of eating disorders. The EAT-26 is one of the most widely used diagnostic instruments in research on eating disorders worldwide.

Interpretation of the EAT-26 questionnaire is based on three “referral criteria,” which determine whether the respondent should undergo further evaluation for eating disorder risk. Section A of the questionnaire consists of 26 items assessing attitudes toward eating, dieting behaviors, and preoccupation with food, reflecting one’s general relationship with eating and body weight. *Section A* of the questionnaire consists of 26 items. Participants rate each item on a six-point scale. Items 1–25 are scored as follows: *Always* = 3 points; *Usually* = 2 points; *Often* = 1 point; all other responses = 0 points. Item 26 is reverse-scored: *Never* = 3 points, etc. The total score ranges from 0 to 78. A score ≥20 indicates an elevated risk of an eating disorder and the potential need for further clinical assessment. *Section B* addresses behavioral patterns that may indicate symptoms of eating disorders or recent significant weight loss. These questions focus on compensatory behaviors such as laxative use, self-induced vomiting, binge eating, excessive physical activity and rapid or substantial weight reduction within a short time. An affirmative response to any of these items may suggest the presence of problematic behaviors and warrant further evaluation. *Section C* contains questions on respondents’ height, weight, and sex, which allow for the calculation of *Body Mass Index (BMI)*. The BMI score may indicate a potential risk of an eating disorder, particularly when body weight is significantly below age adjusted norms. Analysis of BMI in conjunction with other data helps identify individuals at risk and determine the necessity for further diagnostic assessment (21). Table 2 presents BMI thresholds used to determine underweight status depending on age and sex (20).

**Table 2:** Interpretation of men’s and women’s BMI compared with norms for ages (20).

Age	19	20	>20
BMI-male	19.0	19.5	20.0
BMI-female	18.0	18.5	19

### 2.6 Düsseldorf orthorexia scale (DOS-PL)

To assess orthorexic attitudes and behaviors, the *Düsseldorf Orthorexia Scale (DOS)* was employed. This screening tool allows for the rapid and effective identification of individuals at increased risk of orthorexia nervosa (ON). The scale consists of 10 items rated on a 4-point Likert scale: *Strongly disagree* = 1 point; *Disagree* = 2 points; *Somewhat agree* = 3 points; *Strongly agree* = 4 points. The maximum score is 40. A score ≥ 30 is considered indicative of ON, while scores between 25 and 29 reflect risk of its occurrence. In this study, the Polish adaptation of the DOS (PL-DOS) was applied (22).

### 2.7 Body esteem scale (BES)

Body image was assessed using the Body Esteem Scale (BES) in the Polish adaptation by Lipowska and Lipowski (23). The BES comprises 35 items divided into three subscales, which differ for men and women. Responses are given on a 5-point Likert scale, where 1 indicates strong negative feelings, 5 represents strong positive feelings, and 3 reflects neutral attitudes (23).

For men, the subscales include physical attractiveness (PA), upper body strength (UBS), and physical condition (PC). Physical attractiveness is based on features that largely determine the perception of male attractiveness, including facial characteristics and body parts such as hips or feet. Upper body strength evaluates both the size and function of body parts (e.g., arms, chest) as well as perceived strength and capability. Physical condition refers to endurance, agility and overall physical fitness.

For women, the subscales consist of sexual attractiveness (SA), weight control (WC), and physical condition (PC). Sexual attractiveness relates to body parts less amenable to change through exercise (e.g., lips, breasts). Weight control reflects perceptions of body areas that can be modified through diet and physical activity. Physical condition assesses endurance, strength, and agility.

BES raw scores were converted into sten scores using normative tables for age and sex. Based on these, three categories were distinguished: low body esteem (sten 1–3), average (sten 4–7), and high (sten 8–10) (23).

### 2.8 Statistical analysis

Statistical analyses were performed using *Statistica v.13.3* (StatSoft Poland) and *R v.4.0.0* (The R Foundation for Statistical Computing, 2020). Quantitative variables were presented as means ± standard deviations (M ± SD), while qualitative variables were expressed as counts and percentages (%). The distribution of numerical variables was verified with the Shapiro–Wilk test. Depending on data type and distribution, the following tests were applied: *Student’s t-test* for normally distributed comparisons between two groups; *Mann–Whitney U test* for two groups with non-normal distribution; *Analysis of variance (ANOVA)* for three or more groups with normal distribution; *Kruskal–Wallis test* for three or more groups with non-normal distribution; and the *chi-square (χ^2^) test* for associations between categorical variables. Correlations between EAT-26, DOS-PL, and BES scores were assessed with *Pearson’s correlation coefficient*. A *p*-value < 0.05 was considered statistically significant.

## 3 Results

### 3.1 Sample characteristics

The study participants had the following educational backgrounds: higher education (*n* = 29; 52.73%), secondary education (*n* = 24; 43.64%), and lower secondary education (*n* = 2; 3.63%). A total of 9 karate athletes reported chronic diseases: asthma (*n* = 4), hypothyroidism (*n* = 2), chronic sinusitis (*n* = 1), diabetes mellitus (*n* = 1), and glaucoma (*n* = 1). The medications used included symbicort, comboterol, insulin, and euthyrox. The detailed characteristics of the study group are presented in Table 3.

**Table 3:** Characteristics of the study group (*n* = 55).

Variable	Total (*n* = 55)	F (*n* = 33)	M (*n* = 22)	*p*-value
Age (X ± SD)	26.3 ± 6.68	24.7 ± 5.55	28,8 ± 6.6	0.033*
Height (cm) (X ± SD)	172 ± 9.59	166 ± 5.6	181 ± 6.11	<0.001*
Body weight (kg) (X ± SD)	71.1 ± 16,6	64.4 ± 12.7	81.2 ± 11.12	<0.001*
BMI (kg/m^2^) (X ± SD)	23.8 ± 3.39	23.3 ± 3.8	24.6 ± 2.54	0.011*
Weekly training hours (h) (X ± SD)	10.1 ± 3.3	9.48 ± 3.29	11.2 ± 3.12	0.068

F, female; M, male; *p < 0.005.

Based on the calculated BMI of the athletes, 18.18% (*n* = 10) of respondents were classified as overweight, 75.36% (*n* = 42) had a normal body weight and 5.46% (*n* = 3) were classified as obese. None of the athletes were underweight.

Among women, 12.12% (*n* = 4) were overweight, 3.03% (*n* = 1) were obese, while 84.85% (*n* = 28) had a normal body weight. Analysis of BMI values among men showed that 26.09% (*n* = 6) were overweight, 8.70% (*n* = 2) were obese, and 60.87% (*n* = 14) had a normal body weight.

No statistically significant differences were observed in the distribution of BMI between women and men (*p* = 0.181).

A total of 54 participants indicated more than one source of nutritional knowledge. The primary source for all athletes was the internet (*n* = 38), followed by dietitians (*n* = 30), coaches (*n* = 22), scientific literature (*n* = 16), other athletes (*n* = 15), while friends were the least frequently reported source (*n* = 2).

The majority of respondents did not exclude any food groups from their diet (*n* = 36; 65.45%). The remaining participants (*n* = 19; 35.55%) excluded one or several food products. No sex-based differences were observed. The specific products excluded by the respondents are presented in Table 4.

**Table 4:** Food products excluded from the diet of the respondents.

Sex	F (*n* = 33) *n* (%)	M (*n* = 23) *n* (%)	Total (*n* = 55) *n* (%)	*p*-value
Foods excluded from the diet				
No exclusions	20 (66.61)	16 (69.57)	36 (65.45)	0.364
Lactose-containing products	2 (6.06)	1 (4.35)	3 (5.45)	0.827
Red meat	3 (9.09)	0	3 (5.45)	0.156
Fish and seafood	4 (12.12)	0	4 (7.27)	0.097
Gluten-containing products	0	1 (4.35)	1 (1.82)	0.235
Dairy products	1 (3.13)	1 (4.35)	2 (3.64)	0.791
Meat	4 (12.12)	1 (4.35)	5 (9.09)	0.156

F, female; M, male.

A question regarding weight reduction prior to competitions was also included. Nearly half of the athletes (47.47%) reported engaging in weight cutting practices before competitions. However, no statistically significant sex based differences were observed, and detailed information is presented in Table 5.

**Table 5:** Responses to the question: “Do you cut weight to compete in your weight class?” (*n* = 55).

Sex	F (*n* = 33) *n* (%)	M (*n* = 23) *n* (%)	Total (*n* = 55) *n* (%)	*p*-value
“Do you reduce your body weight to qualify for a weight category?”				
Yes	16 (48.48)	10 (43.48)	26 (47.47)	0.835
No	5 (15.16)	6 (26.09)	11 (20)	0.281
No, but I have cut weight in the past	12 (36.36)	6 (26.09)	18 (32.73)	0.492

F, female; M, male.

**Figure 1:**
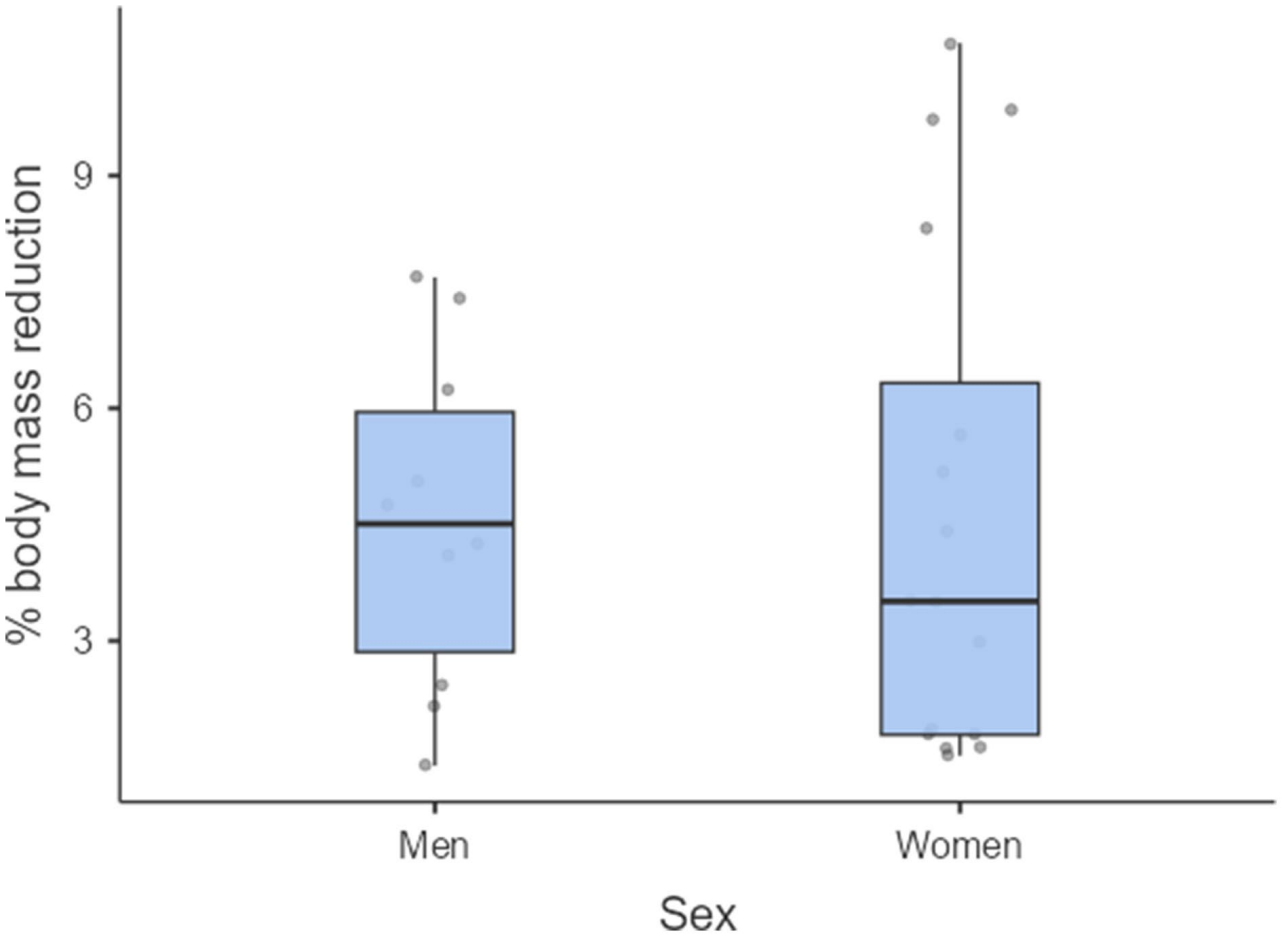
Reduced body mass percentage by sex (F = 33; M = 22).

Among women who cut weight to meet a weight category, the mean body mass reduction was 4.63% (*n* = 16), and among men 4.59% (*n* = 10). Figure 1 presents the sex-specific comparison of percentage body mass reduction. The difference between women and men was not statistically significant (*p* = 0.772).

### 3.2 Risk of eating disorders

Based on the results of the EAT-26 questionnaire, Part A, it was estimated that 12.73% of respondents (*n* = 7), both women and men, are at risk of developing eating disorders. These individuals should consult a specialist for further diagnostic evaluation. The analysis revealed statistically significant differences between women and men in Part A of the EAT-26 test (*p* = 0.021). However, no statistically significant associations were found between nutritional status, as assessed by BMI according to WHO guidelines and the total score of Part A of the EAT-26 test (*p* = 0.289). Similarly, no significant differences were observed between Part A scores and self-reported weight reduction practices in preparation for competitions (*p* = 0.289).

According to the results of the behavioral questions in Part B of the EAT-26 test, it was estimated that 55.58% of women and 54.55% of men met the criteria indicating a potential risk of eating disorders. No significant sex related differences were found in Part B scores of the EAT-26 (*p* = 0.824). Statistical analysis did not reveal significant associations between nutritional status, assessed by BMI values according to WHO guidelines, and the total score of Part B of the EAT-26 (*p* = 0.282). Likewise, no significant differences were found between Part B results and whether respondents engaged in weight reduction practices in preparation for competitions (*p* = 0.327).

No respondents were identified as being at risk of ED in Part C, as all participants demonstrated normative body mass values according to age specific standards.

Based on the overall results and interpretation of the EAT-26 questionnaire, 58.12% (*n* = 32) of respondents (both women and men) met at least one of the three criteria that may indicate the probable presence of or susceptibility to eating disorders. These individuals should be referred to a specialist for further diagnostic evaluation. Differences between sexes were observed only in Part A of the EAT-26 questionnaire (*p* = 0.021). Detailed results are presented in Table 6.

**Table 6:** Risk of eating disorders (EAT-26).

EAT-26	Total (*n* = 55)		F (*n* = 33)		M (*n* = 22)		*p*-value
	No risk	Elevated risk	No risk	Elevated risk	No risk	Elevated risk	
Part A (X ± SD)	48 (87.27)	7 (12.73)	26 (78.79)	7 (21.21)	22 (100)	0	0.021*
Part B (X ± SD)	24 (43.64)	31 (56.36)	14 (89.8)	19 (10.2)	10 (44.45)	12 (54.55)	0.824
Part C (X ± SD)	55 (100)	0	33 (100)	0	22 (100)	0	1
Entire (X ± SD)	23 (41.82)	32 (58.18)	13 (39.39)	20 (60.61)	10 (45.45)	12 (54.55)	0.655

F, female; M, male; *p < 0.05.

### 3.3 Düsseldorf orthorexia scale (DOS-PL)

**Figure 2:**
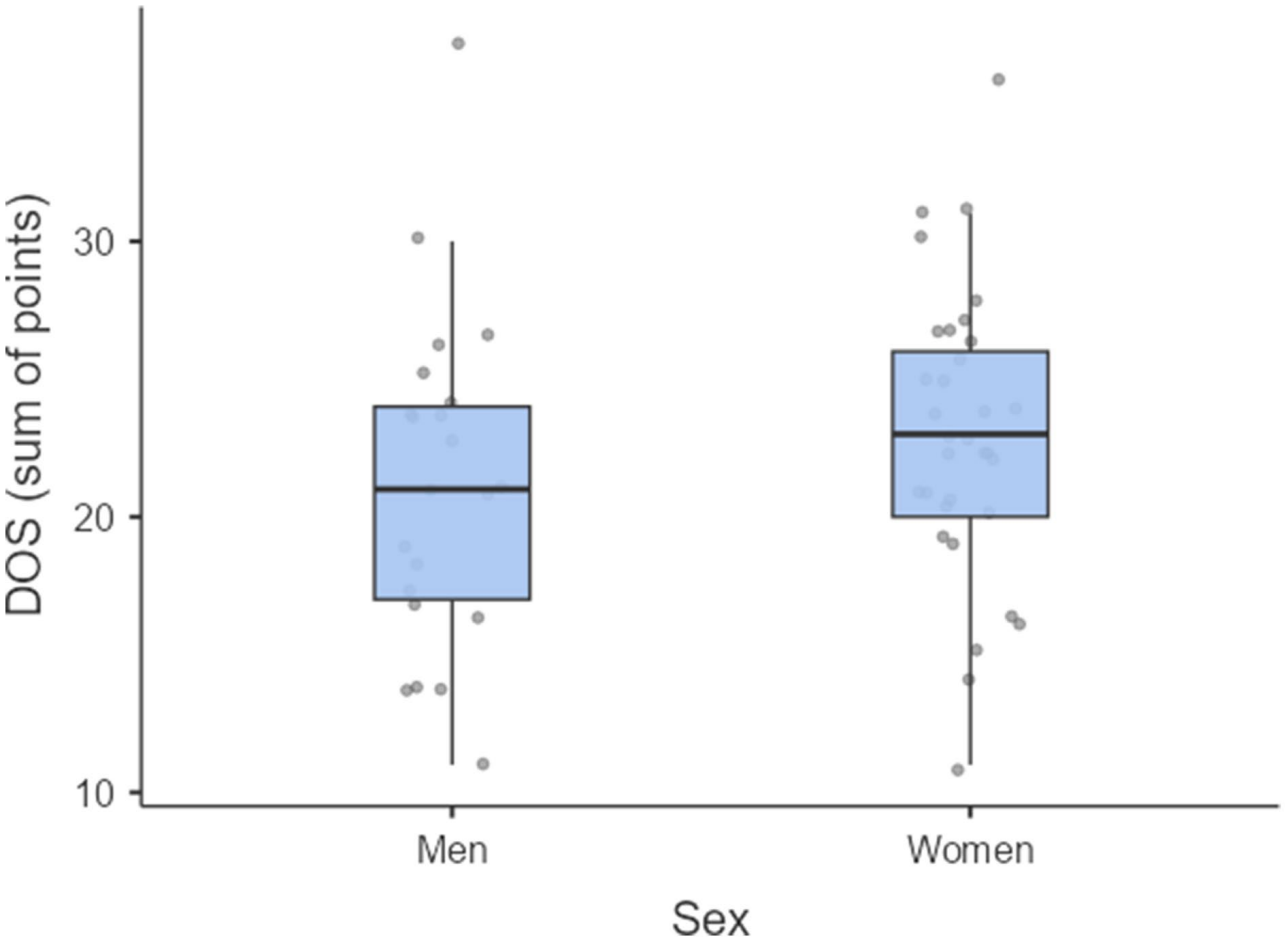
Distribution of DOS-PL total scores among female (*n* = 33) and male (*n* = 22) karate athletes.

Based on the DOS questionnaire, orthorexic behaviors were identified in 6 respondents (10.9%) and 11 participants (20.0%) were at elevated risk of orthorexia nervosa. Figure 2 presents the distribution of DOS-PL total scores by sex. No statistically significant differences were observed (*p* = 0.221).

Table 7 presents the DOS-PL interpretation stratified by sex; no statistically significant differences in the risk of orthorexia nervosa were observed between groups (*p* = 0.546).

**Table 7:** Risk of orthorexia nervosa (DOS-PL) stratified by sex (*n* = 55).

Total (*n* = 55)			F (*n* = 33)			M (*n* = 22)			*p*-value
No risk	Elevated risk	Presence	No risk	Elevated risk	Presence	No risk	Elevated risk	Presence	
38 (69.09)	11 (20.0)	6 (12.91)	21 (63.64)	8 (24.24)	4 (12.12)	17 (77.27)	3 (13.64)	2 (9.09)	0.546

F, female; M, male.

### 3.4 Body esteem scale (BES)

According to the BES analysis, most women obtained low sten scores in physical attractiveness (*n* = 21) and weight control (*n* = 20), whereas the largest number achieved high scores in physical condition (*n* = 15).

Among men, medium sten scores predominated for physical attractiveness (*n* = 13), while the greatest number recorded low scores for upper body strength (*n* = 13) and physical condition (*n* = 9).

Detailed results are presented in Tables 8, 9.

**Table 8:** Body assessment of athletes based on sten scores in the interpretation of DOS among women (*n* = 33).

Sten average	F (*n* = 33)
SA [sten], X ± SD	3.21 ± 2.37
WC [sten], X ± SD	4.91 ± 2.57
PC [sten], X ± SD	4.91 ± 2.57

Assessment of the attractiveness subscale	Low	Medium	High
SA, *n* (%)	21 (63.64)	9 (27.27)	3 (9.09)
WC, *n* (%)	20 (60.7)	9 (27.27)	4 (12.12)
PC, *n* (%)	11 (33.33)	7 (21.21)	15 (45.46)

SA, sexual attractiveness; WC, weight control; PC, physical condition; F, female.

**Table 9:** Body assessment of athletes based on sten scores in the interpretation of DOS among men (*n* = 22).

Sten average	M (*n* = 22)
PA [sten], X ± SD	5.27 ± 2.43
UBS [sten], X ± SD	3.27 ± 1.20
PC [sten], X ± SD	6.36 ± 1.71

Assessment of the attractiveness subscale	Low	Medium	High
PA, *n* (%)	5 (22.73)	13 (59.09)	4 (18.18)
UBS, *n* (%)	13 (59.09)	8 (36.36)	1 (4.55)
PC, *n* (%)	9 (40.91)	8 (36.36)	5 (22.73)

PA, physical attractiveness; UBS, upper body strength; PC, physical condition; M, male.

No significant differences were observed between athlete groups in BMI classification, education level, or self-reported weight reduction for competition preparation with respect to scores across the individual BES categories, in either men or women.

We also examined the association between EAT-26 classification and BES outcomes across all three subscales for both sexes. No significant associations were found between risk of eating disorders and BES subscale scores. Results for men are presented in Table 10 and for women in Table 11.

**Table 10:** The relationship between EAT-26 and BES scores in male athletes (*n* = 22).

EAT-26	Low	Medium	High	*p*-value	V Cramer
PA					
No risk	6 (60.0)	3 (30.0)	1 (10.0)	*p* = 0.576	0.224
Elevated risk	7 (58.33)	2 (16.67)	3 (25.0)		
UBS					
No risk	7 (70.0)	3 (30.0)	0	*p* = 0.495	0.253
Elevated risk	6 (50.0))	5 (41.77)	1 (8.33)		
PC					
No risk	5 (50.0)	3 (30.0)	2 (20.)	*p* = 0.728	0.170
Elevated risk	4 (33.33)	5 (41.67)	3 (25.0)		

PA, physical attractiveness; UBS=Upper Body Strength; PC=Physical Condition.

**Table 11:** The relationship between EAT-26 and BES scores in female athletes (*n* = 33).

EAT-26	Low	Medium	High	*p*-value	V Cramer
SA					
No risk	6 (46.15)	5 (38.47)	2 (15.38))	*p* = 0.229	0.299
Elevated risk	15 (75.0)	4 (20.0)	1 (5.0)		
WC					
No risk	7 (53.84)	3 (23.08)	3 (23.08)	*p* = 0.298	0.271
Elevated risk	13 (65.0)	6 (30.0)	1 (5.0)		
PC					
No risk	5 (38.47)	2 (15.38)	6 (46.15)	*p* = 0.773	0.125
Elevated risk	6 (30.0)	5 (25.0)	9 (45.0)		

SA, sexual attractiveness; WC, weight control; PC, physical condition.

Associations between the total scores of the EAT-26, DOS and the individual BES categories were examined. In the male group, statistically significant positive correlations were observed between UBS and PA (*r* = 0.624; *p* = 0.002), between PC and PA (*r* = 0.629; p = 0.002) and between PC and UBS (*r* = 0.507; *p* = 0.016). This indicates that higher perceived physical attractiveness is accompanied by higher self-ratings of upper body strength and overall physical condition. No significant correlations were found between EAT-26 or DOS scores and any of the BES subscales, suggesting no direct association between risk of eating disorders/orthorexia and body esteem dimensions in men. Detailed data are presented in Table 12.

**Table 12:** Pearson correlation matrix between EAT-26, DOS and AF, SC and PF scales in men (*n* = 22).

Variable	EAT-26	DOS	PA	UBS	PC
EAT-26	–				
DOS	0.187 (*p* = 0.405)	–			
PA	−0.109 (*p* = 0.629)	−0.147 (*p* = 0.515)	–		
UBS	−0.016 (*p* = 0.942)	0.070 (*p*-0.758)	0.624 (*p* = 0.002*)	–	
PC	−0.087 (*p* = 0.702)	0.047 (*p* = 0.836)	0.629 (*p* = 0.002*)	0.507 (*p* = 0.016*)	–

PA, physical attractiveness; UBS, upper body strength; PC, physical condition; *p < 0.05.

In women, strong positive correlations were observed between SA and WC (*r* = 0.723, *p* < 0.001) and between SA and PC (*r* = 0.520, *p* = 0.002), indicating that higher perceived sexual attractiveness is associated with better weight control and physical condition. Weight control (WC) also correlated significantly with physical condition (PC) (*r* = 0.609, *p* < 0.001).

EAT-26 scores showed negative correlations with SA (*r* = −0.381, *p* = 0.029), WC (*r* = −0.549, *p* < 0.001), and PC (*r* = −0.693, *p* < 0.001), meaning that higher self-ratings in these three domains were associated with lower EAT-26 scores.

Additionally, total EAT-26 scores correlated positively and significantly with total DOS scores (*r* = 0.367, *p* = 0.036). The remaining correlation coefficients did not reach statistical significance (*p* > 0.05). Detailed data are presented in Table 13.

**Table 13:** Pearson correlation matrix between EAT-26, DOS and AF, SC, and PF scales in woman (*n* = 33).

Variable	EAT-26	DOS	SA	WC	PC
EAT-26	–				
DOS	0.367 (*p* = 0.036*)	–			
SA	−0.381 (*p* = 0.029*)	−0.246 (*p* = 0.168)	–		
WC	−0.547 (*p* = <0.001*)	−0.296 (*p* = 0.094)	0.723 (*p* = <0.001*)	–	
PC	−0.693 (*p* = <0.001*)	−0.256 (*p* = 0.151)	0.520 (*p* = 0.002*)	0.609 (*p* = <0.001*)	–

SA, sexual attractiveness; WC, weight control; PC, physical condition; *p < 0.05.

## 4 Discussion

This study aimed to assess the risk of eating disorders, tendencies toward orthorexia nervosa and attitudes toward one’s own body in a cohort of competitive karate athletes achieving high performance. The objective was to understand how intense competition, top level performance demands and pressure to maintain specific physical parameters influence psychological and behavioral aspects related to eating and body perception. Interpreting these findings is crucial not only for identifying health risks among karate athletes but also for formulating recommendations regarding psychological support, nutrition education, and preventive strategies to safeguard health and sporting effectiveness. Prior studies have reported a higher prevalence of eating disorders among athletes compared with controls (24–26).

Overall EAT-26 interpretation indicated that 58.12% of respondents met at least one of the three referral criteria suggestive of a probable eating disorder or elevated susceptibility. This highlights the need for specialist consultation and further diagnostic evaluation. Consistent with the literature, such findings are not isolated. Athletes competing in weight-category disciplines appear particularly vulnerable to disordered eating (6, 9, 11). In Rouveix et al. (27), 26% of female judoka were at risk of ED. In the present sample, women scored significantly higher on EAT-26 Part A, suggesting greater vulnerability to negative eating attitudes; this aligns with prior work in which female athletes more frequently reported disturbed eating patterns and stronger appearance related pressures (28, 29). Meyer et al. (28) further noted that women more often than men reported fear of weight gain and adhered to stricter dietary patterns regardless of BMI. Notably, despite these component level differences, no sex differences emerged in the overall EAT-26 score, suggesting that the risk profile may differ by sex while overall magnitude remains comparable.

Using the DOS-PL, 10.9% of participants met the threshold for orthorexic behaviors and 20% were at elevated risk with no significant sex differences. Among 236 climbers assessed with the DOS-PL, 12.7% met ON criteria and an additional 25% were at elevated risk (30). Zydek et al. (31) reported ON behaviors in 9.7% of young football players and elevated risk in 18.3%. These findings underscore the need for further research attention, particularly as ON despite not being recognized in ICD-11 or DSM-5 may substantially impair psychological and social functioning (14–17).

BES results indicated that women more often reported low self-ratings of sexual attractiveness and weight control while simultaneously scoring higher on physical condition. Among men, medium scores predominated for physical attractiveness, but many reported low ratings of upper body strength and physical condition. The link between poor body esteem and ED risk is consistent with prior findings highlighting the central role of body image in ED development (7, 9). In women, all three BES subscales (SA, WC, PC) correlated negatively with the EAT-26 total score, indicating that higher body esteem was associated with lower eating disorder risk. Moreover, EAT-26 total score correlated positively with DOS total, suggesting that orthorexic attitudes may co-occur with classical ED symptoms; similar patterns were reported by Barthels et al. (31). This supports incorporating ON screening into routine assessments among athletes.

In men, no significant correlations emerged between BES and EAT-26 or DOS. However, strong positive associations were observed within BES, especially between physical attractiveness and perceived strength/fitness. This implies that men’s sense of attractiveness may depend more on functional strength and overall fitness than on body mass per se. This resonates with Rouveix et al. (27), who showed that in male combat athletes, physical dominance and conditioning outweigh purely aesthetic body evaluations.

Contrary to common assumptions about the centrality of BMI in ED risk, the present results revealed no significant association between BMI and EAT-26 or DOS-PL. Similarly, Borowiec et al. (32) found BMI was not a significant predictor of ED risk among female athletes from various sports; rather, body dissatisfaction and participation in lean sports were key. These findings emphasize that psychological aspects of body perception may be more consequential than objective somatic parameters in ED development. Importantly, not the absolute BMI value but the frequency and methods of weight reduction may be more relevant: nearly half of the present sample routinely attempted to lose weight before competitions, potentially increasing reliance on unhealthy weight-control practices and elevating ED risk. As noted by Martínez-Rodríguez et al. (6), this pattern aligns with the phenomenon of weight cycling—repeated weight loss and regain—which has been linked to worse physical and mental health outcomes in athletes.

A strength of this study is its focus on a narrowly defined, elite cohort contact karate athletes with at least national championship level achievements capturing a population exposed to pronounced weight-related pressures inherent to weight classes. The use of validated psychometric tools enhances validity and reliability, and the inclusion of multiple psychological variables enables a multidimensional assessment. Limitations of the present study should be acknowledged. First, the research relied on self-reported data, which introduces the possibility of cognitive biases and response tendencies. The cross-sectional design further limits the ability to infer causal relationships between variables. Moreover, several potentially relevant mediators—such as competitive stress, perfectionism, social support, and coach influence—were not assessed. The use of BMI as an indicator of nutritional status represents another limitation, as BMI does not differentiate between fat and lean body mass. In athletic populations, particularly in strength and combat sports, this measure may overestimate the prevalence of overweight or obesity due to increased muscle mass. Future research should employ direct body composition analyses, such as bioelectrical impedance or dual-energy X-ray absorptiometry (DEXA), to obtain more accurate assessments. Additionally, the study did not include some potentially important sociodemographic variables (e.g., income level, place of residence, or family background), which could influence body image and eating behaviors. The sample size was relatively small and drawn exclusively from elite athletes, which limits the generalizability of findings to broader karate or sports populations. Furthermore, because club representatives assisted in selecting participants, there is a potential risk of selection bias—clubs might have preferentially invited athletes with particular attitudes toward nutrition or body image. Finally, data on injury history and perceived external pressure from relatives, sponsors, or other social environments were not collected. Although all participants confirmed the absence of chronic and psychiatric disorders as part of the inclusion criteria, future studies should consider the impact of injuries and psychosocial stressors on eating behaviors and body image in athletes. Moreover, the present study did not include qualitative methods such as in-depth interviews, which could have provided a richer understanding of athletes’ subjective experiences, motivations, and contextual factors influencing their eating behaviors and body image. Future research should consider combining quantitative and qualitative approaches to capture the complexity of psychological and behavioral determinants in this population. Despite these limitations, the present research constitutes the first comprehensive study addressing eating disorder risk, orthorexic tendencies, and body esteem among elite contact karate athletes. It provides a meaningful foundation for further investigations and for developing preventive and educational strategies tailored to weight-category sports.

## 5 Conclusion

This study suggest that competitors in contact karate represent a high risk group for dysfunctional eating attitudes and body image disturbances, potentially facilitating the onset of eating disorders, including orthorexia nervosa. Body image, particularly among women, appears to play a protective or risk enhancing role in shaping eating related behaviors. Pressure to reduce weight before competitions and sport culture-specific physique ideals may exacerbate these difficulties. The findings underscore the need for comprehensive educational, psychodietetic, and preventive initiatives within weight class sports. Early recognition of warning signs and greater awareness among athletes, coaches, and support staff regarding healthy nutrition practices and body acceptance are essential. Further research is warranted to identify causal mechanisms and to develop effective intervention strategies.

## Data Availability

The datasets presented in this article are not readily available due to the confidentiality of the participants. Requests to access the datasets should be directed to dobkowska-szefer@sum.edu.pl.
